# miRNAs in Herpesvirus Infection: Powerful Regulators in Small Packages

**DOI:** 10.3390/v15020429

**Published:** 2023-02-03

**Authors:** Debashree Dass, Kishore Dhotre, Muskan Chakraborty, Anushka Nath, Anwesha Banerjee, Parikshit Bagchi, Anupam Mukherjee

**Affiliations:** 1 Division of Virology, ICMR-National AIDS Research Institute, Pune 411026, India; 2 Department of Cell and Developmental Biology, University of Michigan, Ann Arbor, MI 48109, USA

**Keywords:** *Herpesviridae*, antiviral target, miRNA, vmiRNA, viral latency, RNAi therapeutics

## Abstract

microRNAs are a class of small, single-stranded, noncoding RNAs that regulate gene expression. They can be significantly dysregulated upon exposure to any infection, serving as important biomarkers and therapeutic targets. Numerous human DNA viruses, along with several herpesviruses, have been found to encode and express functional viral microRNAs known as vmiRNAs, which can play a vital role in host–pathogen interactions by controlling the viral life cycle and altering host biological pathways. Viruses have also adopted a variety of strategies to prevent being targeted by cellular miRNAs. Cellular miRNAs can act as anti- or proviral components, and their dysregulation occurs during a wide range of infections, including herpesvirus infection. This demonstrates the significance of miRNAs in host herpesvirus infection. The current state of knowledge regarding microRNAs and their role in the different stages of herpes virus infection are discussed in this review. It also delineates the therapeutic and biomarker potential of these microRNAs in future research directions.

## 1. Introduction

microRNAs (miRNAs) were first discovered in the nematode *Caenorhabditis elegans* in 1993 [1], but were not considered a distinct class of biological regulators until the early 2000s. These are noncoding, small (approximately 22-nucleotide-long) RNAs capable of the alteration of gene expressions [2]. miRNAs are the key regulators in a variety of biological processes, infections, and diseases. Alterations in miRNA expression have been associated with a multitude of diseases [3]. The gene expression regulated by miRNAs is post-translational. miRNAs modulate mRNA translation and degradation through base-pair complementarity. Several viruses encode miRNAs in their genomes. The intricate interaction between host and virus to modulate the miRNA pathway usually promotes viral infection and persistence by lowering immune detection, avoiding apoptosis, boosting cell proliferation, or encouraging lytic or latent infection. According to recent reports, many DNA viruses encode miRNAs. Of those, the human herpes virus encodes the majority of the miRNAs.

Herpesviruses are members of the *Herpesviridae* family (herpein meaning “to creep”). They have a unique structural organization consisting of a long linear dsDNA genome of 100–250 kb encased in an icosahedral capsid with a diameter of 100–110 nm and 162 capsomeres. This is then coated in a layer of amorphous proteins known as the tegument, which is enclosed in a lipid bilayer envelope of roughly a dozen viral proteins and glycoproteins [4].

There are a total of eight human herpesviruses that can be subdivided into three subfamilies: (i) alpha herpesviruses; (ii) beta herpesviruses; (iii) gamma herpesviruses [5]. The alpha herpesviruses target neurons for long-term latency and infect epithelial cells for their transmission through the skin or mucosa. The beta and gamma subfamilies of herpesviruses prefer to infect leukocytes as well as epithelial cells. The replication of herpesviruses occurs in the nucleus, and they use cytoplasmic organelles for the production and maturation of proteins. Herpesviruses leads to latent infections that last a lifetime. The viral latency is achieved by the circularization of the viral genome packed in histone proteins to form an episomal DNA element [6]. In order to avoid immune recognition, it is important to maintain a minimum production of viral proteins. These viruses can be reactivated to cause recurrent or new bouts of disease in their hosts due to the exchange between lytic (productive) and latent (dormant) infections [7].

Viruses are known to modulate the host signaling pathway and immune response. Herpesviruses have evolved a variety of ways to evade the host immune response, such as EBV, which encodes for the viral version of the human IL-10 cytokine. Viral IL-10 affects the cytotoxic effector function of natural killer cells in infected B cells, impedes the function of CD4+ T cells, and dysregulates the cytokine response [8]. Human herpesviruses that encode miRNAs to regulate the expression of their genes, host genes, or both are the herpes simplex virus types 1 and 2 (HSV-1 and HSV-2, respectively), belonging to the alpha-herpesviruses subfamily; human cytomegalovirus (HCMV), from the beta-herpesviruses subfamily; Epstein–Barr virus (EBV) and human herpesvirus 8 (HHV-8), also known as Kaposi’s sarcoma-associated herpesvirus (KSHV), both from the gamma-herpesviruses subfamily [9] (Table 1).

miRNAs play essential roles in regulating pathogen infection, and an increasing number of them are associated with host–pathogen interactions that have been identified in recent years. The dysregulation of miRNA frequently occurs in a variety of diseases. Because of their small size and nonimmunogenic nature, miRNAs are perfect for utilization as regulatory molecules by viruses. As mentioned earlier, a variety of human DNA viruses, including numerous herpesviruses, encode miRNAs. The Epstein occurs Barr virus (EBV) strain B95-8, a member of the herpesvirus family, presented the first characterization of a viral miRNA [14]. Currently, over two hundred vmiRNAs have been found, mostly in the Herpesviridae family. It has been demonstrated that herpes vmiRNAs encourage latent infection by preventing lytic replication of the virus, either directly by targeting the genes involved in viral replication or indirectly by interfering with the host pathways that control the biology of the virus. In this review, we focus on both host and viral miRNAs of herpesviruses and their role in regulating viral infection and pathogenesis. This review summarizes the miRNAs found in herpesviruses and their targets and roles in immune regulation and modulation of virus replication, and talks about the potential of using miRNAs as biomarkers and therapeutics for herpes virus infection.

## 2. General Overview and Synthesis of miRNA

The synthesis of miRNA is restricted to canonical and noncanonical pathways. Canonical synthesis basically refers to the general pathway for the synthesis of miRNA, whereas noncanonical pathways refer to the alternative rather than the conventional process.

### 2.1. Canonical Pathway for miRNA Synthesis

The canonical pathway is the primary pathway by which miRNAs are processed.

#### 2.1.1. Synthesis of Cellular miRNAs

**Figure 1:**
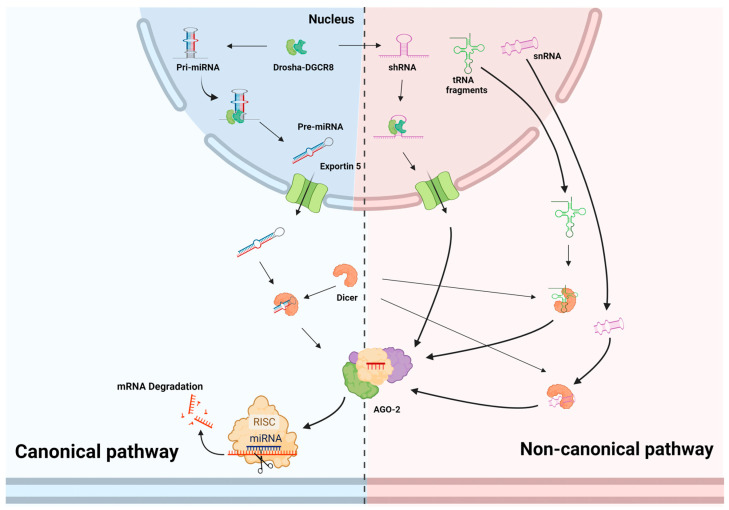
miRNA biogenesis. The canonical and noncanonical pathway of miRNA biogenesis from host and viral genome. In the canonical pathway, the biogenesis of the miRNA begins with synthesis of pri-miRNA transcript. pri-miRNA is processed and cleaved by the Drosha and DGCR8 complex to produce a precursor miRNA (pre-miRNA). Exportin-5 exports the pre-miRNA into the cytoplasm, where the pre-miRNA is further processed by Dicer to produce the mature miRNA duplex. One miRNA strand is loaded into the argonaute (AGO) of the RNA-induced silencing complex (RISC). The noncanonical miRNA biogenesis process bypasses the processing step of the Drosha- or Dicer-dependent cleavage. Ultimately, all the pathways lead to functional-miRNA-induced RISC complex and follow the same procedure as the canonical route.

The expression of several genes is post-transcriptionally regulated by miRNAs. miRNAs contain 21 or 22 nucleotides (which can vary from 19 to 25 nucleotides). Cellular miRNA synthesis can occur through both canonical and noncanonical mechanisms [17]. In the canonical pathway, an active miRNA is generated by a two-step method of nucleolytic processing. RNA polymerase II transcribes pri-miRNA in the nucleus, marking the start of miRNA synthesis. Drosha and Dicer, two RNase III family enzymes, catalyze the conversion of pri-miRNAs into functional miRNAs in two phases [18]. The Drosha and DiGeorge syndrome critical region gene-8 (DGCR8), which together are referred to as the microprocessor complex and are found in the nucleus, mediate the initial stage [19]. Pre-miRNAs, or precursor miRNAs, are cleaved from pri-miRNAs at the initial stage and exported by exportin-5 from the nucleus to the cytoplasm [20]. The second phase is carried out in the cytoplasm, where Dicer further processes pre-miRNAs to produce the active miRNA [21]. Therefore, the stem loop, also known as pre-miRNA, is released from the pri-miRNA by the first RNA split, and the pre-miRNA is then used to generate the mature miRNA by the second split. When miRNAs attach to entirely or partially complementary sequences, translation is inhibited, and mRNA is degraded (Figure 1).

#### 2.1.2. Synthesis of Viral miRNAs

Similar to cellular miRNAs, viral miRNAs or vmiRNAs are also produced in the nucleus by RNA polymerase II transcribed miRNA genes, which results in a mature 21–25 nucleotide dsRNA that is set to be transported onto the argonaute (Ago) complex of proteins, which are the essential components of the miRNA-induced RISC (miRISC), where miRNA–mRNA interaction takes place [22] (Figure 1). To effectively inhibit their target mRNAs, vmiRNAs need to interact with the mRNA to repress it. The interaction is centered on the “seed” region, located at nucleotides 2–8 from the 5′ end [23]. An additional 3′ pairing increases the repression caused by the vmiRNAs. This mRNA repression is accomplished in one of two ways: either by repression of translation or by making mRNAs unstable. According to ongoing research, the two mechanisms occur consecutively, indicating that target mRNA destabilization is the final step in the repression of mRNA [24].

### 2.2. Noncanonical Pathway for miRNA Synthesis

Several noncanonical mechanisms of cellular miRNA biogenesis have been discovered. However, none of them have been described for viral miRNAs. Interestingly, several viruses, such as saimiriine herpesvirus 2 (SaHV-2) and murid herpesvirus 4 (MuHV-4), have evolved miRNA synthesis pathways that do not involve Drosha. The pre-miRNAs of saimiriine herpesvirus 2 and murid herpesvirus 4 are transcribed as small nuclear RNA (snRNA)-pre-miRNAs and transfer RNA (tRNA)-pre-miRNAs, respectively. Pre-miRNA release is accomplished by nucleases after the corresponding pre-miRNAs have been processed by integrator and tRNAse Z, respectively (Figure 1). Mature miRNA biogenesis follows the same procedure as the canonical route [25].

## 3. miRNA and Herpes Virus

There are approximately 300 viral miRNAs known, which are encoded by various virus families, out of which the majority of vmiRNAs are encoded by herpesviruses [26]. Viruses exploit the host miRNA production process to replicate in the nucleus of infected cells. Additionally, vmiRNAs have significant characteristics for modifying cellular gene expression while evading detection by the host immune system [27,28]. There have also been reports of certain orthologs of cellular host miRNAs, in addition to the significant sequence divergence between vmiRNAs. miRNAs provide suitable strategies for viruses to control the expression of viral and cellular genes, and viruses have also created defenses against cellular miRNAs. As a result, certain viruses have developed methods for negatively regulating cellular transcripts by imitating the actions of host miRNAs by offering corresponding miRNA recognition sequences. vmiRNAs play an important role in the complex interactions between hosts and viruses. They frequently support viral persistence in the invading organism through a variety of mechanisms, including preventing cell apoptosis, dodging immune responses, and regulating host and viral genes, which frequently result in the suppression of the viral lytic cycle and the promotion of the latent phase [29].

The two serotypes of herpes simplex virus, types 1 and 2, in particular, both infect people when they come into contact with the oral and vaginal mucosa, respectively. They are the most threatening sexually transmitted infection emergencies. The viruses spread and produce a variety of latent infections in sensory or autonomic neurons close to the main infection site, in addition to the mucosa. The latency-associated transcript (LAT), a non-coding viral RNA, is expressed in HSV during latency [30].

The interaction between the virus and the host is significantly influenced by miRNAs. Certain viral and host miRNAs control how the host reacts to viral infections and how the virus spreads. Herpesvirus-encoded vmiRNAs can target cellular transcripts and pathways that support viral gene expression, replication, and the establishment and maintenance of latency. vmiRNAs can also alter cellular transcription patterns to create a reactivation-friendly environment, enabling an effective response to the correct external cues. As miRNAs have the capacity to target many genes without provoking an immune response, they are the best choice for this job [31].

Herpesviruses have developed a variety of techniques that enable long-term genome maintenance without the need for acute replication or the production of fresh virions. HSV accomplishes this by infecting neural cells, which are thought to survive for the duration of the host and do not divide [7]. Infection can be eradicated if there are no processes that support cell survival, proliferation, and the transmission of viral genomes to freshly split cells. VmiRNAs have been demonstrated to promote the maintenance and growth of latently infected cells, in addition to limiting acute replication.

Herpesviruses have evolved numerous ways to thwart the host defense mechanisms, including methods that employ vmiRNAs as effectors, to enable effective persistent infection. Several vmiRNAs have the ability to lower immune cell activation by preventing the release of cytokines that promote inflammation. The levels of proinflammatory cytokines interleukin (IL-6) and tumor necrosis factor (TNF-α) are reduced when the miR-UL112, miR-US5-1, and miR-US5-2 of HCMV target secretory pathway components. It was also demonstrated that vmiRNAs inhibit the activation of cytotoxic T cells and natural killer (NK) cells. It has been established that HCMV-miR-UL112 specifically targets MHC class-I-related chain B (MICB), a stress-induced ligand that can activate the NK cell cytotoxic response. Notably, miR-UL112 has the ability to suppress MICB expression, which lowers NK cell activation [32].

### 3.1. Role of miRNAs in HSV Infection

Cellular and viral miRNAs both have a significant impact on host–virus interactions by modulating HSV replication. vmiRNAs regulate viral mRNAs, suppress cellular mRNAs, and aid in virus replication to make it easier to avoid the host defense system [33]. The control of HSV replication by host and viral miRNAs is discussed in this section.

#### 3.1.1. Role of Cellular miRNAs in HSV Replication

Host miRNAs have a role in a number of viral defense mechanisms, and HSV infection is no exception. Cellular miRNAs control HSV replication by directly binding to the HSV genome or by causing changes in the host transcriptome that are mediated by the virus. According to studies, miR-199a is an antiviral that impedes HSV-1 by targeting rho GTPase-activating protein 21 (ARHGAP21), necessary for regular Golgi activity and HSV-1 secondary envelopment, and governing multiple pathways important for the successful replication of herpesviruses, such as ERK/MAPK and PI3K/Akt signaling [34,35]. Additionally, during HSV-1 infection, miR-649 is downregulated, which increases the expression of its target, MALT1. This increased expression prevents viral replication by activating the NF-kB signaling pathway. It is interesting to note that miR-649 levels decrease following infection, and this may contribute to restricting HSV-1 reproduction via a negative feedback loop [36]. ICP4, an HSV-1 immediate early (IE) gene, exclusively binds to the miR-101 promoter and inhibits the expression of its target, GRSF1. GRSF1 attaches to the HSV-1 p40 mRNA and promotes viral replication. Therefore, ICP4 induces miR-101, which inhibits HSV-1 replication [37]. Other studies revealed that miR-101 may target the mitochondrial ATP synthase subunit beta (ATP5), which can aid in HSV-1 infection. ATP5B is a proviral factor that is known to encourage HSV-1 proliferation [38]. Therefore, by focusing on a range of host parameters, miR-101 aids in the regulation of HSV-1 replication.

It was noted that miR-23a, which is increased during HSV-1 infection, promotes viral replication by targeting and blocking the interferon pathway in the gene interferon regulatory factor 1 (IRF1), which is involved in the innate antiviral defense of the cell [39]. The host miRNA miR-373 level is significantly elevated by HSV-1 infection by directly targeting IRF1 and negatively suppressing the IFN-I response, which lowers the expression of interferon-stimulated genes (ISGs) [40]. Similarly, miR-132, which promotes viral proliferation by suppressing the production of IFN-stimulated genes, miR-132 is markedly upregulated during HSV-1 and HCMV infections [41].

Therefore, through targeting cellular or viral components, host miRNAs play a significant role in the outcome of HSV replication. By controlling miRNA expression, we may be able to successfully manage HSV infection and suggest a novel anti-HSV therapeutic target.

#### 3.1.2. Role of Viral miRNAs in HSV Replication

**Figure 2:**
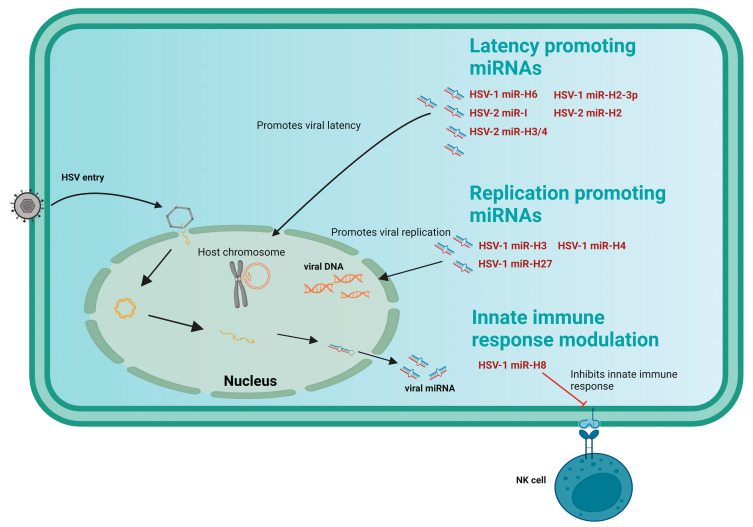
HSV miRNA: A schematic representation of reported miRNAs produced by human simplex virus HSV-1, and HSV-2 to modulate the host immune response by deregulating the pro/antiviral functions, triggering viral replication, and promoting long-term latency by integration/maintaining the viral genome into the host chromosome.

HSV replication can be impacted by viral miRNAs by targeting cellular or viral transcripts (Figure 2). HSV significantly alters host cellular functions, causing substantial manipulations to the transcripts and proteome to facilitate effective viral reproduction. Viral miR-H6 is commonly detected in the course of effective HSV-1 infection. It is known to target the viral infected cell polypeptide (ICP4), an early and late gene trans-activator, in human corneal epithelial cells to inhibit replication [42]. The viral miRNA, miR-H2-3p, is involved in the dysregulation of the cytosolic recognition of viral DNA by targeting the DEAD-box helicase 41 (DDX41) gene, resulting in the attenuation of the innate immune response [43]. Another HSV-1 miRNA, miR-H8, prevents the viral growth and release of several immune-modulating components by targeting the glycosylphosphatidylinositol gene (GPI) [44]. In contrast, miR-H-27 restricts the transcriptional performance of viral IE and early genes by targeting and obstructing Kelch-like 24 (KLHL24). This lessens host cell inhibition, which is beneficial for effective viral reproduction and growth as well as for evading cell-mediated immunity [45] (Table 2).

#### 3.1.3. Role of miRNAs in HSV Latency

The latency-associated transcript (LAT), a noncoding viral RNA, is expressed in HSV during latency (Figure 2) [30]. The viral miRNAs encoded by LAT are engaged in establishing, maintaining, and reactivating viral latency. One such example is seen when HSV-1 miR-H6 targets the polypeptide 4 (ICP4) protein, resulting in the enhancement in lytic infection. ICP4, an IE gene of HSV-1, is observed to promote HSV-1 genes and inhibit LAT activity. Thus, miR-H6 seems to have a crucial role in the latency of HSV-1 [42]. A similar effect is observed when miR-H2 targets the ICP0 protein, an activator of the IE early and late genes of HSV-1 [46]. An example of the combinatorial nature of miRNAs can be seen when two viral miRNAs, miR-H3 and miR-H4, efficiently bind with ICP34.5, which is a lytic neurovirulence factor. These miRNAs, hence, help with establishing and preserving the latent behavior of the virus by functioning as a molecular switch to turn on and off the IE genes while evading host immune surveillance [30]. When the virus is reactivated from a dormant state, HSV-1 releases the miRNAs miR-H28 and miR-H29, which assemble in neurons. These miRNAs prevent the synthesis of viral mRNA and proteins and restrict the size of the plaque [50]. HSV-2 miRNAs elude the immune system similar to HSV-1 due to their common characteristics [51]. Few HSV-2 miRNAs also promote latency and suppression of cellular defense such as HSV-1 miRNAs, due to the structural and functional homology among themselves [12,49,52,53,54]. In HSV-2, miR-H2 and miR-H3/4 contribute to viral latency through silencing ICP0 and ICP34.5, respectively [55,56].

A total of 27 functional miRNA sequences in HSV-1 [10] and 24 in HSV-2 [12] have been identified from the 18 stem loops encoded by HSV-1 and HSV-2, with many of these sequences being shared among both viruses. Although they have the ability to control latency, the bulk of HSV-1 miRNA functions are still unclear. The miRNA sequences of HSV-1 and HSV-2 exhibit a high degree of similarity (about 70%) and play comparable roles. Similar to HSV-1 miRNAs, HSV-2 miRNAs have a role in immune evasion and latency. Regulating cellular gene expression is equally essential for the efficient establishment of viral latency, as is limiting acute replication through viral gene expression regulation.

Along with viral miRNAs, cellular miRNAs can contribute to the initiation, continuation, and reactivation of HSV latency. They include miR-138 and miR-155 [57]. In a recent study, it was reported that host miR-138 promotes HSV-1 latency by targeting the host OCT-1 and FOXC1 genes as well as the viral ICP0 gene [58]. However, during HSV-2 infection, the host miR-138 regulates the OCT-1, FOXC1, and viral ICP0 genes, as well as UL19 and UL20 genes, suggesting that alpha herpesviruses have evolved to exploit the neuronal miRNAs in order to promote viral latency [59]. Similarly, neurons expressing miR-138 target ICP0, the trans-activator of viral lytic gene. In this way, it assists in host survival and HSV-1 latency [47].

Remarkably, herpesviruses have developed methods allowing the establishment of lifelong latency in their host. These interactions exhibit a key role in establishing and maintaining latency.

#### 3.1.4. Immunopathological Consequence of HSV Infection

Ocular herpes: Globally, HSV-1 ocular infections are reported to be the foremost cause of corneal blindness. Ocular herpes manifests as conjunctivitis, blepharitis, and dendritic keratitis, causing necrotizing stromal keratitis and disciform stromal edema [60]. Recurrence of infections leads to herpetic stromal keratitis (HSK), an immunopathological disease causing blindness [61]. A distinctive feature of HSK is progressive scarring in the corneal stroma.

There are several miRNAs involved in immune function and the pathogenesis of HSK [62]. For example, the overexpression of the proinflammatory miR-155 results in the enhancement in Th-1 and Th-17 cell responses. miR-155 knockout mice are immune to developing HSK [63]. Similarly, the upregulation of miR-132 during HSK in the cornea contributes to angiogenesis. Inhibiting its expression effectively diminished angiogenesis and reduced HSK severity [64]. However, the role of other miRNAs in HSK pathogenesis requires more research.

Genital herpes: HSV-2 continues to spread and can result in recurring and severe genital sores [65]. Several individuals who test positive for HSV-2 do not have a record of genital herpes that was clinically relevant. These asymptomatic individuals are the primary transmitters of the virus. Several studies have revealed the vital role of T cells in genital herpes infections. HSV-2-specific T-helper and T-cytotoxic cells continue to infiltrate healed genital herpes lesions. Genital biopsy specimens from humans suffering from recurrent HSV-2 infections show a high concentration of local virus-specific CTLs upon viral clearance [66]. miRNAs play an important role in the pathogenesis of HSV-2-induced genital herpes. In HSV-2-mediated genital herpes infection, miR-592, miR-1245b-5p, miR-124, miR-150, miR-1245b-3p, and miR-342-5p levels are elevated, resulting in epigenetic alterations [67].

Neonatal herpes: More than 50% of neonatal herpetic infections are caused by HSV-1, whereas HSV-2 appears to be responsible for up to 70% of newborn herpes [68]. When HSV-2 is shed from the genitalia during labor and delivery, the relative risk of viral transmission intensifies almost 300 times. A mother’s immune response is important in the control of the virus and clearance of infected tissues; however, an overactive cellular immunity within the delicate environment of the placenta can cause immunopathology. This can lead to dysfunction of the placenta, injury of the fetus, fetal sequelae, and/or miscarriage [60]. However, research on the effect of the maternal T-cell response and the role of miRNA dysregulation on newborn and neonatal herpes is of prime importance.

### 3.2. miRNAs in Human Cytomegalovirus Infection

Belonging to the *Betaherpesvirinae* subfamily, human cytomegalovirus (HCMV) establishes latency upon acute infection, causing increased morbidity and mortality in the immunocompromised. Primary HCMV infection in pregnant women can be perinatally transferred, leading to severe birth defects. Apart from regulating viral genes, HCMV miRNAs can influence cellular genes in order to influence viral latency and replication. HCMV miRNAs are scattered throughout the virus genome as single miRNAs or tiny clusters [69].

**Figure 3:**
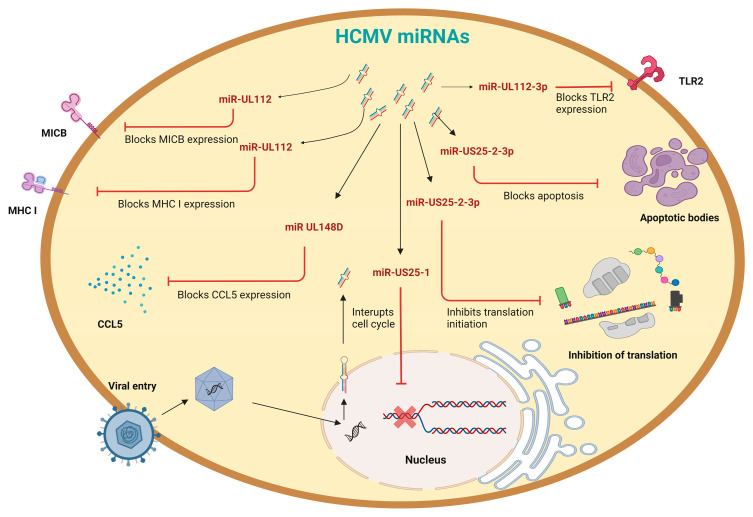
HCMV miRNA: A model representing the functional role of miRNAs produced by the human cytomegalovirus (HCMV) during its active replication cycle. Cellular receptors allow the virus to enter the cell, and the capsid then translocates to the nucleus, where it releases its genome. The lytic phase of HCMV infection produces miRNAs targeting multiple proteins in order to deregulate cellular signaling, gene expression, apoptosis, and innate immune response.

To date, 21 HCMV-originated miRNAs have been discovered, which are generated from 14 stem-loop precursors [13]. HCMV miRNAs target several host genes and pathways to dodge the host immune system to control its cell cycle. HCMV-miR-UL112 assists in the reduction in the recognition of the natural-killer group 2 member D (NKG2D) by targeting the MHC class-I-related chain B (MICB) (Figure 3) [32]. In addition, it acts in tandem with cellular miRNA, miR-376a, to suppress MICB expression, and, eventually, cell death mediated by natural killer cells. This shows how HCMV has evolved to cooperate with cellular miRNAs and target the highly conserved sequences of the 3′ UTR of cellular mRNAs. Another HCMV miRNA, miR-US25-2-3p, targets the tissue inhibitors of metalloprotease 3 (TIMP3), which increases the shedding of MHC class-I-related chain A (MICA), reducing the recognition of NK cells [70]. HCMV-miR-UL112-3p inhibits NF-κB signaling, evading the immune response by targeting TLR2, whereas miR-US4-1 achieves the same by targeting the endoplasmic reticulum aminopeptidase 1 (ERAP1) mRNA, affecting the antigen processing and presentation by MHC class I to cytotoxic T cells [71,72]. Furthermore, by specifically targeting the chemokine (C-C motif) ligand 5 (CCL5) gene, HCMV miRNA UL148D aids in evading the immune response. Because the CCL5 protein promotes NK-cell activation and expansion [73], miR-UL148D, together with miR-UL112 and miR-US25-2-3p, plays a role in HCMV NK evasion.

HCMV-miR-US25-1 interrupts the cell cycle by targeting up to 20 cellular transcripts, including the collagenase stimulatory factor (CD147), cyclin E2 (CCNE2), and BRCA1/BRCA2-containing complex-subunit 3 (BRCC3) [13]. The levels of eukaryotic initiation factor 4A1 (eIF4A1), an RNA helicase that initiates translation, are downregulated by miR-US25-2-3p, possibly promoting latency and cell-cycle control [74]. HCMV-miR-US25-1 targets the V0 subunit C pseudogene 1 (ATP6V0CP1) protein, an ATPase necessary in acidifying endosomal compartments. The regulation of this miRNA may be vital for controlling viral replication and avoiding the host immune system during the latent phase of the virus. HCMV-miR-UL112-1 targets the immediate early trans-activator, IE72, which is vital for the replication of viral DNA [75]. Additionally, miR-UL112-1 expresses the antisense form of the viral uracil DNA glycosylase (UL114), which promotes the production of both early and late viral transcripts [76]. Hence, when miR112-1 is expressed, viral replication is reduced, and viral latency is favored (Table 3).

HCMV miRNA expression, like other viruses, varies with latent and lytic infection. miR-UL112-3p and miR-US22-5p are the most abundant miRNAs in the latent phase [13,77,78]. On the other hand, those upregulated upon lytic infection are miR-US25-1-5p, miR-US25-2-5p, and miR-US29-5p, which are dramatically downregulated during the establishment of infection and latency. Interestingly, the amounts of miR-US25-1-5p, miR-US25-2-5p, and miR-UL112-3p considerably increase once the lytic infection is reactivated. Furthermore, miR-US29-5p was observed during lytic infection, but its presence was negligible during latency. The opposite occurred with miR-US29-3p, demonstrating a mechanism associated with the transition to latency.

Human cancer is influenced by HCMV during its onset and spread. According to some reports, glioblastoma multiforme (GBM), a severe malignancy of the central nervous system, has been linked to HCMV. A three-fold increase in miR-UL112-3p expression was observed in GBM tissues. It was discovered that miR-UL112-3p targets tumor suppressor candidate 3 (TUSC3), a putative tumor suppressor gene, proving that miR-UL112-3p may function as a tumor regulator by specifically targeting TUSC3 in GBM. As a result, miR-UL112-3p might be crucial for the development of GBM [78]. Ulasov et al. examined the relationship between hcmv-miR-UL70-3p and the activation of glioblastoma malignancy [79]. The authors found that hcmv-miR-UL70-3p was 10 times more abundant in tumor tissue than in healthy tissue, indicating that this viral miRNA may have a role in the development of GBM. Moreover, increased miR-UL70-3p expression improves the ability of cancer cells to migrate and invade. Therefore, miR-UL70-3p might encourage the growth and spread of tumors [79].

HCMV alters the expression of the host miRNAs known to possess antiviral functions to favor viral replication. Low levels of hsa-miR-21 were associated with defects caused by congenital HCMV infection in newborns [80]. On the contrary, hsa-miR-124, which inhibits macrophage activation, was overexpressed during HCMV latency, creating a suitable environment in the host cell for the HCMV.

HCMV infection is complicated by to the interactions between the host and virus, which make it persistent lifelong. The precise roles and mechanisms of miRNAs in infection are still being unraveled.

### 3.3. miRNAs in Epstein–Barr Virus (EBV) Infection

EBV, like the other members of its family, can establish latency and persist lifelong in its host. It is known to cause infectious mononucleosis, which commonly affects adolescents and young adults. Following its infection, EBV can transform benign cells to cancerous ones. The cases of undifferentiated nasal NK/T-cell lymphoma, nasopharyngeal carcinoma, post-transplant lymphoma, sporadic and endemic Burkitt’s lymphoma (BL), some subtypes of Hodgkin’s disease, gastric carcinoma, and diffuse large B-cell lymphoma all have high rates of EBV infection [81].

**Figure 4:**
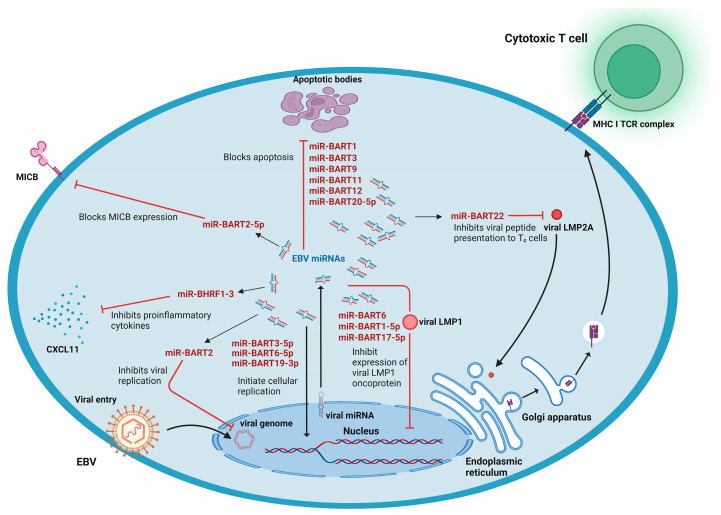
EBV miRNA: Function of Epstein–Barr virus (EBV)-derived miRNAs in the modulation of host signaling pathway and immune evasion. EBV-derived miRNAs downregulate the gene expression of host proteins involved in the process of apoptosis to restrict programmed cell death, tumor suppressor genes to initiate cellular replication, and proinflammatory cytokines to deregulate the pattern recognition receptors. EBV-derived miRNAs also restrict the expression of viral oncoprotein (LMP1) and inhibit viral LMP2A protein synthesis to escape immune recognition.

EBV encodes 25 pre-miRNAs, which give rise to 44 mature miRNAs. EBV miRNAs have three clusters: BHRF1, BART-cluster 1, and BART-cluster 2 [82]. EBV miRNAs miR-BART1-5p, miR-BART16, and miR-BART17-5p, belonging to BART-cluster 1, suppress viral antigen LMP1 [83]. An increase in the levels of EBV-miR-BART22 correlated with a reduction in LMP2 levels, providing strong evidence that it targets LMP2A [84]. EBV-miR-BHRF1-3 evades the host immune system and modulates its defense mechanisms by targeting CXCL11, a chemokine that attracts T cells [85]. Another EBV miRNA, miR-BART2-5p, evades the immune system by targeting MICB and reducing cell recognition (Figure 4) [86]. Furthermore, miR-BART15 was observed to bind the cellular NLRP3, which helps in producing proinflammatory cytokines such as IL-1β and IL-18, providing another method to avoid the immune response by expressing miRNAs [87].

vmiRNAs play a vital part in evading apoptosis. PUMA, a proapoptotic factor (p53-upregulated modulator of apoptosis), is altered by miR-BART5-5p, whereas miR-BART16 and miR-BART1-3p target caspase 3 [88,89]. Many EBV-encoded miRNAs, which are a part of BART-cluster 1, regulate BCL2 interacting mediator of cell death (BIM), such as miR-BART1, miR-BART3, miR-BART9, miR-BART11, and miR-BART12 [90]. Furthermore, the viral miR-BART16 targets translocase of outer mitochondrial membrane 22 homologs (TOMM22), and miR-BART20-5p controls the expression of BCL2-associated death promoter (BAD) protein [90,91]. Additionally, miR-BART6-5p was observed to target Dicer mRNA, whose downregulation may play a role in EBV reactivation [92].

According to various studies, EBV miRNAs can also play a crucial role in the development of cancer. The tumor-suppressor gene deleted in cancer 1 (DICE1) is targeted by miR-BART3-5p, and WNT inhibitory factor (WIF1 1) is targeted by miR-BART19-3p [93,94]. In the case of gastric carcinoma, the expressions of the members of the let-7 family and hsa-miR-200 were highly reduced, many of them being tumor-suppressing miRNAs [95]. In diffuse large B-cell lymphoma, miR-BART7, miR-BART22, and miR-BART10 were upregulated, with cellular miRNAs including miR-26b, miR-27b, miR-199a, miR-223, miR-378, miR-424, and miR-23a/b being highly expressed, whereas miR-20b, miR-29b/c, miR-151-3p, miR-221, miR-222, and miR-106 were underexpressed. miR-BART5 and miR-BART7-5p were upregulated to their maximum in cells from NK/T-cell lymphoma [96]. Almost all of these miRNAs have been linked to cancer etiology, supporting their function in EBV oncogenesis [97].

Several cellular miRNAs are seen to regulate EBV latency and lytic infection. miR-200b and miR-429 target ZEB proteins to modulate EBV reactivation [98]. miR-let-7a regulates EBV reactivation, whose induction by EBNA1 promotes EBV latency [99] (Table 4).

### 3.4. miRNAs in Kaposi’s Sarcoma-Associated Herpes Virus (HHV-8/KSHV) Infection

KSHV or human herpesvirus 8 (HHV-8) belongs to the *Gammaherpesvirinae* and is known to cause several diseases, such as Kaposi’s sarcoma, multicentric Castleman’s disease, and primary effusion lymphoma, especially in immunosuppressed patients. It establishes latent infection in monocytes, dendritic cells, endothelial cells, and B lymphocytes.

KSHV produces 25 mature miRNA sequences from 13 precursor miRNAs that are irregularly dispersed throughout the genome [55]. KSHV miRNAs target cellular mRNAs for the modulation of cytokine responses and the evasion of the immune system. For example, miR-K12-9 and miR-K12-5 target interleukin-1 receptor-associated kinase 1 (IRAK1) and myeloid differentiation primary response 88 (MYD88), respectively, which mediate TLR/IL-1R signaling, reducing inflammation [103]. Moreover, miR-K12-11 attenuates type I interferon signaling by targeting IκB kinase epsilon (IKKε) [104]. The virus encodes for miR-K12-1, which targets MICB mRNA and evades NK-cell recognition [86]. The same viral miRNA, miR-K12-1, interferes with the host protein IκB and the cyclin-dependent kinase inhibitor p21, which promote cell-cycle advancement and aid in cell survival, respectively [105]. Similarly, miR-K1-10 targets TWEAKR, protecting the cells from TWEAK-induced apoptosis, and interferes with the transformation of growth factor beta receptor II (TGFBR2), influencing the TGF-β pathway [106,107]. SMAD family member 5 (SMAD5), a TGF-β pathway intermediate, is targeted by miR-K12-11 [108]. TGF-β pathway inhibition leads to cell survival and virally induced oncogenesis. Other vmiRNAs, miR-K12-1, miR-K12-3, and miR-K12-4-3p, suppress caspase-3 in cooperation and inhibit caspase-3-induced apoptosis [109]. Various KSHV miRNAs are associated with promoting infection by increasing the susceptibility of host cells to viral infection. Among those, miR-K12-1, miR-K12-9, and miR-K12-11 increase the expression of xCT (a viral fusion-entry receptor), enhancing the susceptibility of macrophages and endothelial cells to KSHV. miR-K12-11 is known to target BTB and CNC homology 1 (BACH-1), a negative regulator of xCT [110,111,112]. miR-K12-11 and miR-K12-6 work together to reduce the expression of the cellular transcription factor v-maf avian musculoaponeurotic fibrosarcoma oncogene homolog (MAF), which again downregulates xCT [113,114].

**Figure 5:**
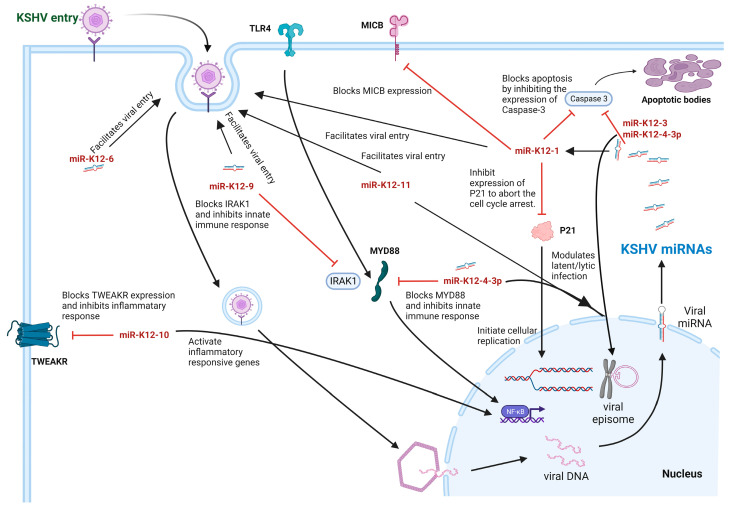
KSHV miRNA: Kaposi’s sarcoma-associated herpesvirus (KSHV)-encoded miRNAs exhibit dysregulation of host signaling pathways. The viral miRNAs block apoptosis by inhibiting the gene expression of caspase-3 protein, modulating latent/lytic infection by facilitating viral genome integration into the host chromosome and aborting the cell-cycle arrest by inhibiting the P21 protein synthesis to initiate cellular replication. KSHV miRNA blocks MYD88 activity, thereby inhibiting the innate immune response against the virus. Several miRNAs are reported to facilitate viral entry by modulating expression of several host proteins.

KSHV miRNAs also promote latency by targeting viral and host mRNAs. miR-K12-7 and miR-K12-9 target the IE gene ORF50, reducing replication and transcription activator (RTA) expression, which is encoded within this gene [115]. Additionally, miR-K12-4 and miR-K12-3 modulate the expression of RTA by targeting RBL 2 and NFIB, respectively, ultimately contributing to the viral latent phase [116,117]. Finally, miR-K12-1 aids in latency by targeting IκBα, leading to an NF-κB-dependent latent period [107]. Contrarily, vmiRNAs are observed upon the induction of the lytic cycle, which is required for infection spread. BCL2-associated transcription factor 1 (BCLAF1) is targeted by miR-K12-5 and miR-K12-9; IKKε is repressed by miR-K12-11, leading to lytic infection (Figure 5) [104,118]. However, their overall mechanisms remain to be revealed.

Dysregulation of host miRNAs may give rise to KSHV-associated cancers, immune regulation, and modulation of virus replication. Two such miRNAs, miR-21 and miR-31, assist in cell migration and angiogenesis, playing an important role in cancer development [119]. Remarkably, KSHV was observed to negatively regulate miR-30b/c and those from the miR-221/miR-222 cluster. All of these host miRNAs have already been recognized as tumor-suppressor genes or oncogenes in various cancer types [120]. Therefore, it would seem that the KSHV-mediated dysregulation of these host miRNAs is crucial for viral cancer progression. KSHV-encoded miRNAs employ different mechanisms to regulate cellular miRNAs and cytokine expression to target host cell survival and establish a successful infection, all while evading the host’s immunity (Table 5).

## 4. miRNAs as Potential Biomarkers

The role of miRNAs as potential disease biomarkers was first explored in 2008, when Lawrie et al. showed that patterns of miRNA expression were altered in patients with diffuse large B-cell lymphoma (DLBCL) [122]. Since then, studies have been conducted to investigate the efficacy of miRNAs as biomarkers for a variety of diseases, including Alzheimer’s [123], diabetes [124], many human malignancies, and a variety of no-infectious disorders. An acceptable biomarker should be readily available, unique, and sensitive to the disorder and can be discovered and assessed using minimally invasive techniques [125].

miRNAs can be easily retrieved from urine, blood, and other body fluids (saliva and urine) using next-generation sequencing (NGS), microarray profiling, real-time PCR arrays, and liquid biopsies [126,127]. miRNAs are also highly specific to the tissue or cell, and their concentration levels greatly vary with the progression of the disease. This property can be used in the differentiation of cancer stages and to measure the responsiveness of the patient to the therapy. Unique viral miRNAs encoded in different diseases could prove promising to differentiate between viral diseases that exhibit similar clinical symptoms. The fact that miRNAs detected within exosomes in plasma outlive mRNAs in vivo gives them another benefit when used as indicators; they are more stable because of their encapsulated miRNAs when analyzed as samples, making them more efficient to assess ongoing infections or herpesvirus-induced cancers [128]. Moreover, miRNAs that are linked to argonaute proteins may be delivered into the extracellular environment, limiting degradation. As a result, miRNAs are protected from nuclease breakdown. These elements combine to make miRNAs appealing as novel biomarkers [129].

### 4.1. miRNAs as Potential Biomarker during Herpes Virus Infection

Herpesvirus miRNAs could be highly potent biomarkers in the prognosis of disease, tracking its advancement and guiding decisions to provide suitable therapy. For example, patients who receive organ transplantation are likely to be infected with HCMV, and this can greatly affect organ rejection, morbidity, and mortality. Currently, acute infection can be tested by examining IgM/IgG titers, the HCMV pp65 antigenemia assay, and quantitative nucleic acid testing (QNAT), specifically PCR. However, IgM antibodies can take up to weeks to appear, and false-positive results are possible in patients with EBV or HHV-6 infections. Furthermore, the pp65 antigenemia assay cannot be used in patients with neutropenia, and QNAT is extremely sensitive and quick but cannot differentiate between viral shedding during active disease and that of postdisease clearance [130]. Some HCMV miRNAs could be utilized to track infection or reactivation, as they can be differentially expressed at various points of the viral life cycle. Kawano et al. observed that the levels of some EBV miRNAs in plasma were raised during active chronic EBV infection when contrasted with a control group of patients suffering from infectious mononucleosis [131]. Similarly, comparing the miRNAs expressed during lytic and latent infection may also be useful in the detection, assessment, and treatment of disease.

### 4.2. Limitations of miRNAs as Biomarkers

Unfortunately, research into miRNAs as biomarkers is still in its early stages. The reports of different teams that have examined the same disease profiles differ, so the results are not reproducible. To address this issue, extra technical competence and standardization must be established for the earliest stages of the operation, such as the collection of samples, transportation, and storage, as well as the analysis of data for the methodologies used. Additionally, the expression of certain miRNAs in people from different backgrounds and their conservation between different strains of a particular virus need to be thoroughly studied.

## 5. Therapeutic Considerations

Antiviral drugs can inhibit viral replication, but they have no impact on latent viral DNA. miRNAs can be utilized as therapeutics for viral infections and their resultant pathologies. Knowing the biological functions of particular miRNAs has enabled us to consider them as alternative treatments or potential therapeutics. Clinical uses of miRNAs are essential, as miRNA-focused suppression may have a significant therapeutic influence on virus evolution and tropism modulation in vivo. Thus, it is mandatory to appreciate and acknowledge the complex interplay between viral replication, viral miRNAs, and host-cell miRNAs. In an miRNA-based therapeutic strategy, the miRNA expression, which is altered in different diseases, is corrected by injecting the downregulated miRNAs or by inhibiting the upregulated ones. Antisense miRNAs that have been chemically transformed to have improved durability are used in locked nucleic acid (LNA) or antagomirs administered in nanoparticle form as present therapeutics to downregulate miRNA expression [132,133]. miRNA mimics are similarly used to restore miRNA function.

One of the key issues in miRNA delivery in vivo is the degradation by nucleases before reaching the target. To overcome this limitation, several chemical modifications have been tried, which may lead to decreased miRNA activity, off-target effects, and toxic metabolite production due to the degradation of these molecules. To protect the miRNAs from these conditions, a suitable delivery system is required, which will ideally provide efficient target binding delivered by a nonimmunogenic carrier. Nonetheless, therapeutics based on RNAi have faced their challenges, including the design of the delivery system, antisense oligonucleotide biostability, delivery of the drug to the desired cell/organelle, and off-target effects. However, novel approaches are being developed to overcome these initial drawbacks and discover better therapeutics. Miravirsen (LNA), which targets miR-122 during HCV infection, is an intriguing example of an miRNA-based antiviral treatment currently being developed in chimpanzees [134]. Another study on mice discovered that intranasally administering five chemically modified miRNA mimics could target the viral RNA, suppress H1N1 replication, and safeguard the mice from infection [135].

## 6. Future Perspectives

Amid the hype surrounding miRNAs, their effectiveness is constrained by their ineffective targeting, rapid circulation, and off-target consequences of medicines based only on miRNAs. miRNA-loaded nanoparticles (NPs) have been presented as a solution to these obstacles [136,137,138]. The loaded agent, such as miRNA, can be protected by nanoparticles from the surrounding environment, minimizing inactivation or degradation and increasing circulation time and selective accumulation. Furthermore, using miRNAs for therapeutic purposes has significant benefits over more conventional approaches such as gene therapy, which deliver larger molecules such as mRNA or DNA. First, because a single miRNA may control numerous mRNAs at once, miRNAs have a wider spectrum of therapeutic targets. The activity of siRNAs and mRNAs is restricted to the suppression or overexpression of a single particular gene, respectively, but miRNAs in the form of miRNA inhibitors and miRNA mimics can affect both the expression and suppression of many genes. Additionally, miRNAs are small, which may make it easier for them to be delivered into cells and to operate at the cytoplasmic level. Due to their small size, they can also be efficiently encapsulated into nanoparticles [139,140]. Because naked miRNAs are prone to quick breakdown and conventional transfection methods are unsuitable for in-human applications, the advanced and safe delivery of miRNAs is essential for their use in clinics [141]. These delivery difficulties can be solved by encapsulating miRNAs into NPs, which improves targeting effectiveness and minimizes the off-target impacts of the enclosed cargo [142].

## 7. Conclusions

Viruses need host-cell mechanisms for their replication. Therefore, they have intricate interactions with their hosts, which make it difficult to target the virus without causing damage to the host cells. Fighting the virus by targeting its specific proteins and pathways has been successful against some viruses, but with unavoidable host-cell damage/toxicity. As a result, there are extremely few effective antivirals available. Hence, the potential of RNAi has been used as a defense mechanism.

miRNAs have a better prospect of being indicators of infection. Any exposure to parasites, bacteria, and viruses alters these molecules in body fluids. miRNA expression affects and modifies the output of host genes. Therefore, the new era of screening methods is now focused on biomarkers such as miRNAs. Presently, miRNA-based therapy is still in its early stages, and multiple aspects must be tackled before it can be safely and reliably used in the clinical setting. miRNAs are thought to be the next generation of therapeutic molecules, serving as indicators and regulators of various diseases and infections after siRNAs. However, the precise function and mechanism of host miRNAs in safeguarding against viruses have so far remained obscure. Because of the upcoming trends in miRNA detection systems and the introduction of commonly available miRNA-based diagnostics, miRNAs will be used as a highly powerful and crucial component in diagnostics and even as curative agents. miRNAs are projected to shortly enter the clinical arena as effective disease biomarkers and therapeutics, paving the way for one of the most inventive and hopeful prospects in modern medicine.

## Figures and Tables

**Table 1:** Human-herpesviruses-encoded miRNAs ^1^.

Virus Family	Virus Species	Number of Known Pre-miRNAs	Number of miRNAs	Citations
**α herpesviruses**	Herpes simplex virus 1	16	27	[5,10,11]
	Herpes simplex virus 2	18	24	[5,12]
**β herpesviruses**	Human cytomegalovirus	14	21	[13]
**γ herpesviruses**	Epstein–Barr virus	25	44	[14]
	Kaposi’s sarcoma-associated herpesvirus	13	25	[15,16]

^1^ The table represents the number of pre-miRNAs and miRNAs encoded by different herpesviruses.

**Table 2:** Potential cellular and viral targets of HSV miRNAs ^2^.

Herpesvirus	miRNA	Target	Function	References
**Viral targets**				
**HSV-1**	miR-H2-3p	ICP0	Immune evasion	[11,46,47]
	miR-H3, miR-H4	ICP34.5	Immune evasion	[9]
	miR-H6	ICP4	Immune evasion	[9]
	miR-H8	GPI	Inhibits viral growth	[44]
**HSV-2**	miR-I	ICP34.5	Immune evasion	[16,48,49]
	miR-H2	ICP34.5	Immune evasion	[48,49]
	miR-H3/4	ICP0	Immune evasion	[46,48,49]
**Cellular targets**				
**HSV-1**	miR-H-27	KLHL24	Immune evasion, viral replication and proliferation	[45]

^2^ The table presents HSV-encoded vmiRNAs, their targets (cellular/viral), and their modes of action. Abbreviations: ICP0, infected cell polypeptide 0; ICP34.5, infected cell polypeptide 34.5; ICP4, infected cell polypeptide 4; GPI, glycosylphosphatidylinositol; KLHL24, Kelch-like 24.

**Table 3:** Potential cellular and viral targets of HCMV miRNAs ^3^.

HCMV	miRNA	Target	Function	Reference
**Cellular** **targets**	miR-UL112	MICB	Immune evasion	[32]
	miR-US25-2-3p	TIMP3	Immune evasion	[70]
	miR-UL112-3p	TLR2	Immune evasion	[72]
	miRNA UL148D	CCL5	Immune evasion	[73]
	miR-US4-1	ERAP1	Immune evasion	[71]
	miR-US25-1	CD147, CCNE2, EID1, BRCC3, MAPRE2, H3F3B, ATP6V0CP1	Interrupts cell cycle	[11]
	miR-US25-2-3p	eIF4A1	Immune evasion	[74]
**Viral targets**	miR-UL112-1	IE72, UL114	Favors latency	[75]

^3^ The table presents HCMV miRNAs, their targets (cellular/viral), and their modes of action. Abbreviations: MICB, MHC class-I-related chain B; TIMP3, tissue inhibitors of metalloprotease 3; TLR2, Toll-like receptor 2; CCL5, chemokine (C-C motif) ligand 5; ERAP1, endoplasmic reticulum aminopeptidase 1; CD147, collagenase stimulatory factor; CCNE2, cyclin E2; EID1, EP300 interacting inhibitor of differentiation 1; BRCC3, BRCA1/BRCA2-containing complex-subunit 3; MAPRE2, microtubule-associated proteins, RP/EB family, member 2; H3F3B, histone proteins; ATP6V0CP1, ATPase-V0 subunit C pseudogene 1 protein; eIF4A1, eukaryotic initiation factor 4A1; IE72, immediate early trans-activator.

**Table 4:** Potential cellular and viral targets of EBV miRNAs ^4^.

Herpesvirus	miRNA	Target	Function	Reference
**Cellular** **targets**	miR-BHRF1-3	CXCL11	Immune evasion	[85]
	miR-BART1 miR-BART3	BIM	Inhibits apoptosis	[90]
	miR-BART1-3p	CASPASE 3	Inhibits apoptosis	[89]
	miR-BART2-5p	MICB	Immune evasion	[100]
	miR-BART3-5p	DICE1	Cell transformation and proliferation	[93]
	miR-BART5-5p	PUMA	Immune evasion	[88]
	miR-BART6-5p	DICER	Cell transformation and proliferation	[92]
	miR-BART9 miR-BART11 miR-BART12	BIM	Inhibits apoptosis	[90]
	miR-BART15	NLRP3	Immune evasion	[87]
	miR-BART16	TOMM2 CASPASE 3	Immune evasion Inhibits apoptosis	[89,90,101]
	miR-BART19-3p	WIF1	Cell transformation and proliferation	[94]
	miR-BART20-5p	BAD	Inhibits apoptosis	[101]
**Viral** **targets**	miR-BART16, miR-BART17-5p miR-BART1-5p	LMP1	Immune evasion	[83]
	miR-BART22	LMP2A	Immune evasion	[84]
	miR-BART2	BALF5	Regulates viral replication	[102]

^4^ The table presents EBV miRNAs, their targets (cellular/viral), and their modes of action. Abbreviations: CXCL11, C-X-C motif chemokine 11; BIM, BCL2-interacting mediator of cell death; MICB, MHC class-I -elated chain B; DICE1, deleted in cancer 1; PUMA, p53-upregulated modulator of apoptosis; NLRP3, NLR family, pyrin-domain-containing 3; TOMM2, translocase of outer mitochondrial membrane 22 homologs; WIF1, WNT inhibitory factor 1; BAD, BCL2-associated death promoter protein; LMP, latent membrane protein.

**Table 5:** Potential cellular and viral targets of KSHV miRNAs **^5^**.

Herpesvirus	miRNA	Target	Function	References
**Cellular** **targets**	miR-K12-1	MICB P21 IκBα CASPASE 3 xCT	Immune evasion Oncogenesis Cell survival Modulates latent/lytic infection Facilitates viral entry	[105,107,109,110,111,121]
	miR-K12-3	CASPASE 3 NFIB	Cell survival Modulates latent/lytic infection	[109,117]
	miR-K12-4-3p	CASPASE 3 RBL	Cell survival Modulates latent/lytic infection	[109,116]
	miR-K12-5	MYD88 BCLAF1	Immune evasion Modulates latent/lytic infection	[103,118]
	miR-K12-6	xCT	Facilitates viral entry	[110,113]
	miR-K12-9	IRAK1 BCLAF1 xCT	Immune evasion Modulates latent/lytic infection Facilitates viral entry	[103,109,118]
	miR-K12-10	TWEAKR TGFBR2	Immune evasion Cell survival Cell survival Oncogenesis	[106,107]
	miR-K12-11	xCT IKKε SMAD5	Facilitates viral entry Modulates latent/lytic infection Immune evasion Cell survival Oncogenesis	[104,108,110,113]
**Viral targets**	miR-K12-7 miR-K12-9	KSHV ORF50	Modulates latent/lytic infection	[115]

^5^ The table presents KSHV miRNAs, their targets (cellular/viral), and their modes of action. Abbreviations: MICB, MHC class-I-related chain B; P21, cyclin-dependent kinase inhibitor 1A; IκBα, nuclear factor of kappa light polypeptide gene enhancer in B-cells inhibitor, alpha; xCT, solute carrier family 7 (anionic amino acid transporter light chain, xC-system), member 11; NFIB, nuclear factor I/B; RBL, retinoblastoma-like protein 2; MYD88, myeloid differentiation primary response 88; BCLAF1, BCL2-associated transcription factor 1; IRAK1, interleukin 1 receptor-associated kinase 1;TWEAKR, tumor necrosis factor-like weak inducer of apoptosis receptor; TGFBR2, transformation of growth factor beta-receptor II; IKKε, IκB kinase epsilon; SMAD5, SMAD family member 5; KSHV ORF50, Kaposi’s sarcoma-associated herpesvirus open reading frame 50.

## Data Availability

Not applicable.
